# Hyperhomocysteinemia is Associated with Inflammation, Bone Resorption, Vitamin B12 and Folate Deficiency and MTHFR C677T Polymorphism in Postmenopausal Women with Decreased Bone Mineral Density

**DOI:** 10.3390/ijerph17124260

**Published:** 2020-06-15

**Authors:** Massimo De Martinis, Maria Maddalena Sirufo, Cristina Nocelli, Lara Fontanella, Lia Ginaldi

**Affiliations:** 1 Department of Life, Health and Environmental Sciences, University of L’Aquila, 67100 L’Aquila, Italy; maddalena.sirufo@gmail.com (M.M.S.); lia.ginaldi@cc.univaq.it (L.G.); 2 Allergy and Clinical Immunology Unit, Center for the diagnosis and treatment of Osteoporosis, AUSL 04, 64100 Teramo, Italy; 3 Pneumology Unit, AUSL 04, 64100 Teramo, Italy; cristina.nocelli@aslteramo.it; 4 Department of Legal and Social Sciences, University of Chieti-Pescara, 65127 Pescara, Italy; lara.fontanella@unich.it

**Keywords:** osteoporosis, hyperhomocysteinemia, inflammation, vitamin D, bone, vitamin B12, folate, MTHFR, postmenopausal women, bone mineral density

## Abstract

Osteoporosis is an age-related bone disease, affecting mainly postmenopausal women, characterized by decreased bone mineral density (BMD) and consequent risk of fractures. Homocysteine (Hcy), a sulfur-aminoacid whose serum level is regulated by methylenetrahydrofolate reductase (MTHFR) activity and vitamin B12 and folate as cofactors, is a risk factor for inflammatory diseases. Literature data concerning the link between Hcy and osteoporosis are still debated. The aim of our study was to assess the relationship among Hcy and BMD, inflammation, vitamin status and bone turnover in postmenopausal osteoporosis. In 252 postmenopausal women, BMD was measured by dual-energy X-ray absorptiometry (DXA). In addition to serum Hcy, erythrocyte sedimentation rate (ESR), C-reactive protein (CRP) and bone turnover markers (bone alkaline phosphatase-BAP, osteocalcin-OC, C-terminal telopeptide of type I collagen (CTX), vitamin deficiencies and MTHFR-C677T polymorphism were evaluated. Hcy, inflammation, bone resorption markers and prevalence of C677T polymorphism were higher, whereas vitamin D, B12, folate, and bone formation markers were lower in women with decreased BMD compared to those with normal BMD. Our results suggest a significant association between Hcy, BMD and inflammation in postmenopausal osteoporosis. The regulation of Hcy overproduction and the modulation of the inflammatory substrate could represent additional therapeutic approaches for osteoporosis prevention.

## 1. Introduction

Osteoporosis is an age-related skeletal disease characterized by decreased bone mineral density (BMD) and bone structure deterioration resulting in increased risk of fractures [1]. It represents an important public health problem, as it is one of the main causes of disability and mortality in the elderly [2]. Osteoporosis affects mainly postmenopausal women, due the estrogen decline [3], but many other contributing factors, such as genetic and metabolic disorders, lifestyle, environmental and inflammatory factors could intervene [4,5,6,7]. In addition to the search for effective antiosteoporotic drugs [8,9,10], also the identification of new osteoporotic risk factors that can be modified is of paramount importance. Beside the well-known classic risk factors [1], recent studies have suggested that also elevated homocysteine (Hcy) serum levels may represent a risk factor for osteoporosis and skeletal fractures [11].

Hcy is a sulfur amino acid, which is formed following the loss of a methyl group by the essential amino acid methionine. Hcy is then metabolized, and vitamin B12 and folate play an important role as cofactors in this process. The enzyme 5-methylenetrahydrofolate reductase (MTHFR), involved in the metabolism of folate, is essential for the remethylation of Hcy to methionine, through the intervention of vitamin B12 [12]. The serum concentration of Hcy, under physiological conditions, is between 5 and 12 µmol/L. An alteration in the pathways of metabolism of Hcy determines a condition of hyperhomocysteinemia.

Both genetic and acquired hyperhomocysteinemia can cause important pathological changes in the body [13]. The most common genetic defect that can cause hyperhomocysteinemia is the MTHFR gene C677T polymorphism: the prevalence of such homozygotes in Europe can reach 20% while the frequency of heterozygotes is calculated as 30–40%, resulting in a reduction of the corresponding enzyme that can be responsible for a slight to moderate hyperhomocysteinemia [14]. Another common condition which can cause an increase in serum Hcy levels is vitamin B12 and/or folate deficiency, which prevents the correct process of remethylation from Hcy to methionine [15,16]. Moreover, both aging and menopausal estrogen deficiency are accompanied by an increase in blood Hcy levels [17].

It is well established that hyperhomocysteinemia represents an independent risk factor for age-related chronic inflammatory diseases, including Alzheimer and cardiovascular diseases [18]. Osteoporosis shares the same inflammatory background with these conditions [19]. Moreover, a compromised bone health is observed in patients with homocystinuria, a rare autosomal recessive disease characterized by very high Hcy serum levels. In particular, a low BMD is common in both children and adults with homocystinuria, as assessed by bone densitometry [20]. Further evidence of a possible role of Hcy in osteoporosis comes from the observation of Hcy, vitamin B12 and folate plasma level association with increased risk of fractures [21,22]. For these reasons, a role of Hcy in the pathogenesis of osteoporosis has been postulated, suggesting that hyperhomocysteinemia may be responsible for decreased BMD, bone deterioration, and osteoporotic fractures. However, literature data concerning the link between Hcy and osteoporosis are still debated [23,24,25].

Biochemical markers of bone turnover are divided into markers of bone formation and bone resorption. Bone formation markers are products of active osteoblasts produced during the new bone synthesis. They include the osteoblast enzyme bone-specific alkaline phosphatase (BAP) and the matrix protein osteocalcin (OC). Markers of bone resorption are formed during the degradation phase of bone remodeling, such as the collagen degradation product C-terminal telopeptide of type 1 collagen β cross laps (CTX). All these markers can be measured in the blood.

Vitamin Dplays a central role in regulating bone homeostasis andrepresents another important marker of bone health. Moreover, it is also involved in the direct regulation of cystathionine beta-synthase, a central enzyme in Hcy metabolism.Reduced serum levels of vitamin D may therefore lead to elevated Hcy levels, which in turn may lead to altered bone quality [16].

The aim of our study was to assess the relationship among Hcy, inflammation, B vitamin status and bone turnover in postmenopausal osteoporosis. To this purpose, we measured the Hcy serum level; inflammation indexes, such as erythrocyte sedimentation rate (ESR) and C-reactive protein (CRP); and bone turnover markers in postmenopausal osteoporotic women and healthy controls.To evaluate bone metabolism, we measured the bone resorption marker CTX, as well as BAP and OC as markers of bone synthesis [26]. In addition, we also searched for vitamin D, B12 and folate deficiencies and MTHFR gene C677T polymorphism, which can lead to elevated Hcy levels in the blood [14,22].

## 2. Materials and Methods

Two-hundred and fifty two postmenopausal women, aged 50–65 years (mean age 62 years), among those observed on an outpatient basis at the Center for Osteoporosis of L’Aquila University at the Hospital of Teramo, were recruited in the study. As previously described [27,28], subjects were selected on the basis of a careful clinical examination and medical history, to rule out any co-morbidities and/or therapies that could influence bone resorption [29]. BMD measurements were performed by using dual energy X-ray absorptiometry (DXA) (Hologic QDR 4500 W machine) at the lumbar spine and hip. BMD values were expressed as T-score, i.e., the difference between the BMD value of the examined subject and the mean value in the healthy reference population. Diagnosis of osteoporosis was made according to the World Health Organization criteria [30,31]: a reduction in BMD, indicative of osteoporosis and/or osteopenia, is defined by T-score values lower than −1.5 (osteoporosis <−2.5 and osteopenia between −1.5 and −2.5), whereas T-score values above −1.5 indicate a normal BMD. 

Laboratory parameters were collected from all subjects. Serum Hcy concentrations were measured by using a chemiluminescence immunoassay method (ARCHITECT Homocysteine Reagent Kit–Abbott Diagnostics) [32]. Collected data also included inflammation indices, such as CRP and ESR and serum concentrations of vitamin D, B12 and folate, analyzed by chemiluminescence immunoassay systems (ADVIA Centaur^®^ ReadyPack™ assays, Siemens Healthcare GmbH, Erlangen, Germany) and bone turnover biomarkers such as OC, BAP (Chemiluminescence immunoassay “CLIA”-DiaSorin LIAISON^®^ test, DIASORIN via crescentino snc—Saluggia (Vc), Italy) and CTX (Electrochemiluminescence immunoassay “ECLIA”–ElecsysCobas analytics Roche Diagnostics CH—Basel, Switzerland.). The coefficients of variation (CV) in these methods for the overall reliability of our immunological test results were less than 10%. MTHFR gene C677T polymorphism (MTHFR-C677T), searched by Real Time PCR (MTHFR C677T Mutation Creative Biogene Real Time PCR Kit), was also evaluated. The kit contains a specific ready-to-use system for the detection of MTHRF gene site C677T Mutation in whole blood sample by polymerase chain reaction (PCR) in the real-time PCR system, including reagents and enzymes for the specific amplification of the site C677T Mutation gene DNA. Fluorescence is emitted and measured by the real time systems’ optical unit during PCR.

Clinical history, physical examination and instrumental analysis (spine radiography and vertebral morphometry to highlight any asymptomatic osteoporotic fractures) were also performed in each subject.

The present study was approved by the Internal Review Board of the University of L’Aquila, Italy (ex “Comitato etico di Ateneo” D.R. n. 206/2013 modified D.R. n. 46/2017), and conducted in accordance with the 1975 Helsinki Declaration and its subsequent amendments. Written informed consent was obtained from all participants.

The statistical analysis was performed with SPSS for Windows (version 17.0, IBM, Armonk, NY, USA). Data were expressed as means ± standard deviations (sd) and percentages, as appropriate. Differences between groups were analyzed by Student’s t-test for unpaired data and comparison between two proportions. Although most of the variables considered in the analysis are not normally distributed as testified by Shapiro–Wilk test, it is still possible to apply the parametric Student’s T test as, according to the Central Limit Theorem, the sample mean will be approximately normally distributed for large samples (*n* > 30) as the ones considered in the comparison.Correlation between variables was evaluated by Pearson’s correlation test. The level of statistical significance was set at *p* ≤ 0.05. 

## 3. Results

**Figure 1:**
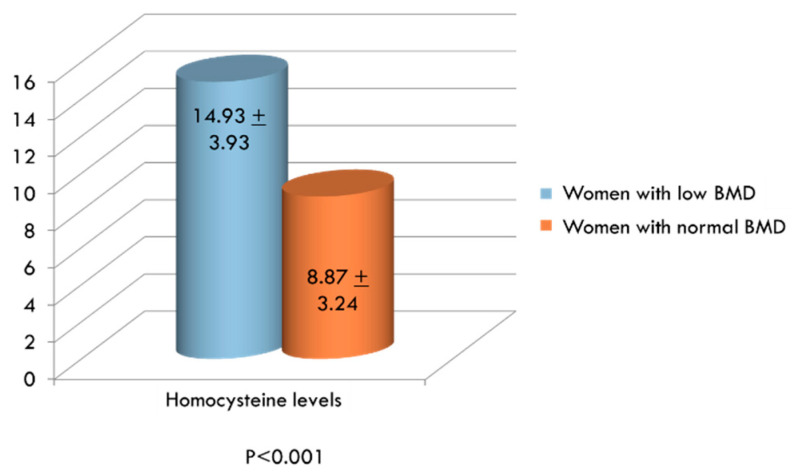
Serum levels of Homocysteine (Hcy, µmol/L) in women with low and normal bone mineral density.

All women were divided into two groups, based on the T-score values: 155 women with low BMD and 97 women with BMD in the normal range. We compared the levels of Hcy in the two groups; as shown in Figure 1, serum Hcy levels were significantly higher in women with osteopenia/osteoporosis than in women with normal BMD.

**Figure 2:**
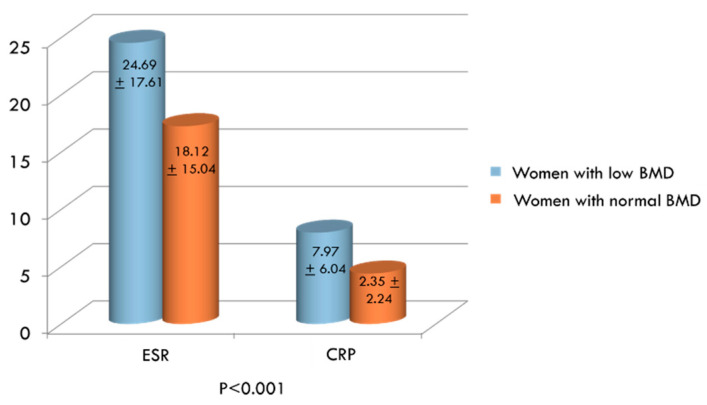
Mean values of inflammatory markers (erythrocyte sedimentation rate (ESR) and C-reactive protein (CRP)) in women with low bone mineral density (BMD) compared to women with normal BMD.

**Figure 3:**
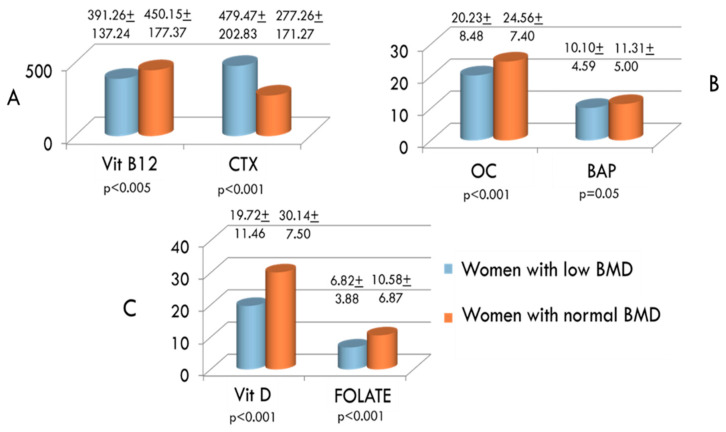
Serum concentrations of vitamin B12 and C-terminal telopeptide of type I collagen (CTX) (**A**), osteocalcin (OC) and bone-specific alkaline phosphatase (BAP) (**B**), and vitamin D and folate (**C**) in women with low and normal BMD.

**Figure 4:**
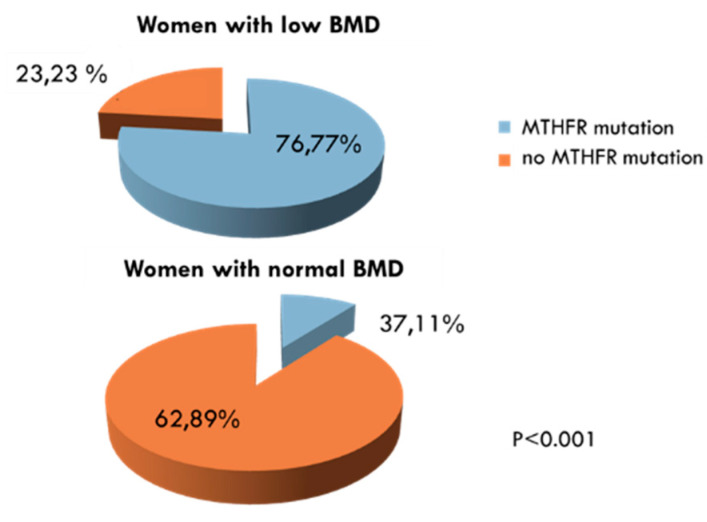
Prevalence of methylenetrahydrofolate reductase (MTHFR)gene C677T polymorphism (MTHFR mutation) in women with low bone mineral density (BMD) compared to women with BMD in the normal range.

In addition to Hcy, inflammation indexes, bone resorption markers, vitamin D, vitamin B12, folate and the possible presence of MTHFR genetic mutation were also evaluated in the two groups. ESR and CRP were significantly higher in women with low BMD compared to women with normal BMD (Figure 2). On the contrary, serum levels of vitamin D, vitamin B12 and folate were significantly lower in women exhibiting decreased BMD than in women with normal BMD values (Figure 3A,C, respectively). Concerning bone remodeling markers, CTX was significantly higher (Figure 3A), whereas both OC and BAP were significantly lower (Figure 3B) in the group of women with decreased BMD compared to women with BMD in the normal range. Since measuring OC and CTX both together is considered as a reasonable choice for evaluating bone degradation and synthesis, we also calculated OC/CTX ratio for each subject. In women with decreased BMD, the OC/CTX ratio was significantly lower than in women with normal BMD (mean 0.0868 ± 0.104 standard deviation vs 0.1399 ± 0.136; *p* < 0.001).Furthermore, the prevalence of the MTHFR mutation among women with decreased BMD is significantly higher than in women with normal BMD (Figure 4).We considered the C677T polymorphism in both heterozygous and homozygous state; of the total women studied, 118 (47%) had MTHFR-C677T polymorphism, and 21 of these (18%) were homozygous.

**Figure 5:**
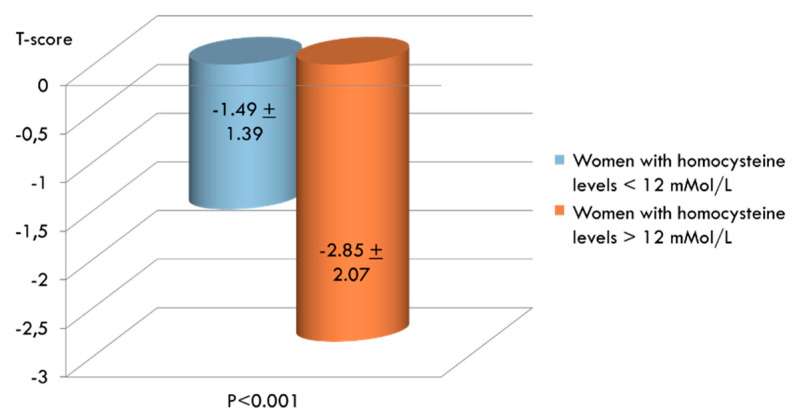
Comparison between mean T-score values in women with normal (n. 105) and increased (n. 147) levels of serum homocysteine (Hcy).

We also divided women by serum Hcy levels and evaluated mean BMD values in the two groups. As shown in Figure 5, mean T-score values were significantly higher in women with serum Hcy levels below 12 µmol/L than in women with serum Hcy levels greater than 12 µmol/L.

**Figure 6:**
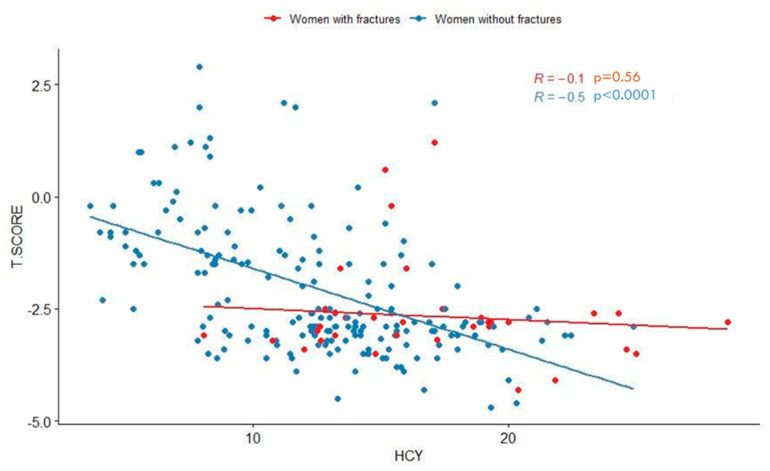
Scatter plot showing significant negative correlation between homocysteine (Hcy) levels and BMD (T-score values) in postmenopausal women. Pearson’s correlation coefficients (R) and *p*-values related to unfractured (blue dots) and fractured (red dots) women are reported.

Moreover, we stratified women according to their BMD and serum Hcy level, also considering the presence or absence of fractures. Thirty-one out of 155 women (20%) with osteopenia/osteoporosis and 3 out of 97 (3%) with normal BMD had vertebral fractures on radiography with spinal morphometry or reported previous femoral or wrist fractures (*p* < 0.001). As shown in Figure 6, whichillustrates how many patients with increased Hcy levels also show decreased T-scores and suffer from fragility fractures, there is a significant negative correlation between homocysteine (Hcy) levels and BMD (T-score values) in postmenopausal women (R = −0.51; *p* < 0.0001). However, considering only fractured patients, the statistical significance is lost. All fractured patients with normal BMD had elevated serum Hcy levels.

All data concerning laboratory findings of postmenopausal women with hyperhomocysteinemia and women with normal Hcy levels are summarized in Table 1. In women with higher Hcy serum levels, the inflammation indexes (ESR and CRP), the serum levels of CTX and, as expected, the prevalence of the MTHFR mutation were significantly higher than in women with normal serum Hcy levels. Conversely, in the latter, both OC, vitamin D, vitamin B12 and folateserum levels were significantly higher compared to women with hyperhomocysteinemia. Moreover, none of the women with the MTHFR mutation and normal serum Hcy levels had a homozygous condition for the MTHFR-C677T mutation.

## 4. Discussion

In this study, we showed the relationship of Hcy with BMD, inflammation and B vitamin status among postmenopausal women. We found that high Hcy and low B vitamin serum concentrations are associated with decreased BMD and increased bone loss and inflammatory indexes. Postmenopausal women with low BMD exhibited higher serum Hcy levels than postmenopausal women with normal BMD. Women with higher Hcy levels expressed lower T-score values, as well as an increase in inflammation and bone resorption markers, compared to women without hyperhomocysteinemia, while markers of new bone formation decreased. The findings of an increased prevalence of the MTHFR gene mutation and a decrease in serum levels of folate and vitamin B12 in women with decreased BMD further support the role of Hcy in the development of postmenopausal osteoporosis. Our results therefore suggest that Hcy, together with inflammation, plays an important role in bone metabolism and is strongly involved in bone resorption leading to decreased BMD in postmenopausal women.

MTHFR C677T has a high degree of heterogeneity in its world distribution. In a recent meta-analysis, the global frequency of T allele was found to be 24.0%. The C677T polymorphism shows a wide regional and ethnic variation. It has been found to have high frequencies in Europeans and American Caucasian population, and the prevalence rises in Mediterranean and Hispanic people, whereas it is less prevalent in Africa and intermediate among Asian populations [33]. The reason for this varied distribution seems to be directed not only by environmental effects (in particular folate and B12 rich/deficient diets), but it is also due to diversity in the various ethnic groups residing in the world. Comparing the observed prevalence of both hyperhomocisteinemia and MTHFR C677T polymorphism with other populations, we found that our results are in line with data from epidemiological studies on larger samples. According to the results from an epidemiological study by Zappacosta et al. [34], estimating MTHFR C677T allele frequency of 50.5% (95% C.I. 43.7–57.3) in Italy, we found a prevalence of 47% in our sample. C677T polymorphism, both homozygous and heterozygous, is responsible for reduced activity of the enzyme, thus inducing hyperhomocysteinemia, especially when associated with low plasma vitamin B12 and folate levels.

The prevalence rates of hyperhomocysteinemia are also highly variable worldwide, ranging from 5–30% in the general population to more than 60% among those with vascular and inflammatory diseases [35]. The prevalence of hyperhomocysteinemia increases with age and is higher in males but significantly increases in women after menopause [36]. Normal levels range very widely in different populations due to special lifestyle factors, such as smoking habit, diet, exercise, alcohol consumption and coffee intake. Other factors include vitamin deficiency, chronic illnesses and drugs, such as antihyperlipidemics and proton pump inhibitors, all of which are commonly observed as age progresses. Hyperhomocysteinemic subjects in our study also showed vitamin B12 and folate deficit. Therefore, besides genetic and environmental features, both age and postmenopausal condition could explain the higher prevalence of hyperhomocysteinemia in our study population, even regardless of the presence of detectable genetic mutations. Because it is thought to be modifiable, hyperhomocysteinemia may represent a clinically important risk factor of osteoporosis.

The association between Hcy and the onset and progression of osteoporosis has been variously addressed by previous clinical reports. Some of them reported an association between higher serum Hcy levels and increased risk of fragility fractures and/or decreased BMD in the elderly [11,37], whereas other studies reported no significant relationship between lumbar spine or femur BMD and serum Hcy levels in postmenopausal women and did not find any significant effect of Hcy on bone metabolism [23,24]. Therefore, data from the literature are controversial, partially depending on the heterogeneity among studied populations. Furthermore, most of the studies did not simultaneously investigate the status of inflammatory and bone turnover markers, as well as the levels of B vitamins and the presence of MTHFR mutations [38].

Postmenopausal osteoporosis recognizes various risk factors in addition to hormonal deficit, from the inflammatory state up to vitamin deficiencies, genetic and metabolic factors. The recent discoveries in the field of osteoimmunology have now clarified the role of chronic inflammation in the pathogenesis of osteoporosis [19]. Hcy is now emerging as another important risk factor for osteoporosis development, since it plays an important role in bone metabolism, both directly and through the synergy of inflammatory processes and nutritional deficits. In accordance with recent studies [39,40], we hypothesize a combined role of inflammation and Hcy in the pathogenesis of osteoporosis in postmenopausal women. The serum level of Hcy is increased in postmenopausal women and an association of hyperhomocysteinemia with increased fracture risk has been suggested [41]. Hcy and inflammation are linked by complex mechanisms of mutual enhancement in postmenopausal osteoporosis [42]. Hcy is a facilitator of chronic inflammation and is elevated in age-related diseases [43]. Hyperhomocysteinemia is even considered an independent risk factor for cardiovascular, neurodegenerative and autoimmune diseases [44]. In addition, these age-related chronic inflammatory diseases are frequently associated with osteoporosis of which they share a common inflammatory background [45]. This is clearly evident in postmenopausal osteoporosis in which the levels of both Hcy and inflammatory markers are particularly high [19,46].

Deficiency of vitamin D is a well-known risk factor for osteoporosis, and like Hcy, vitamin B12 and folate, it plays important roles in bone remodeling. Vitamin D deficiency is also associated with inflammation and hyperhomocysteinemia, and low vitamin D levels are related to several chronic inflammatory diseases [47]. As the primary role of vitamin D is to maintain bone integrity, low levels induce increased bone turnover and reduced bone mineralization. An association between Hcy, vitamin D and BMD in postmenopausal females has been demonstrated [16]. Hypovitaminosis D is associated not only with an increase in biomarkers of bone turnover but also with inflammation and hyperhomocysteinemia. Hypovitaminosis D is associated not only with an increase in biomarkers of bone turnover but also with inflammation and hyperhomocysteinemia.

In addition to the well-known bone protective and anti-inflammatory effect of vitamin D, also vitamin B12 and folate are involved in both inflammation [48] and bone turnover. Interestingly, they are also determinants of Hcy serum concentration [49]. In line with our observation of increased Hcy levels in postmenopausal osteoporosis, we also detected significantly decreased serum concentrations of folate and vitamin B12 in women with low BMD, confirming the results from other authors [50]. Elevated serum Hcy may be the consequence of inadequate folate and vitamin B12 intake. B vitamins have been shown to associate with decreased BMD and higher osteoporotic fracture risk, and vitamin B12 deficiency has also been proposed as independent risk factor for low BMD [51]. However, studies on the effect of supplementation with vitamin B12 and/or folate have had conflicting results [25], further reflecting the complexity of the dynamic process of bone remodeling and the multifactorial postmenopausal osteoporosis pathogenesis.

The mechanisms by which serum Hcy levels are involved in the development of postmenopausal osteoporosis are likely to be multifactorial, involving the mutual interaction of various factors, such as inflammation, increased bone resorption and vitamin B12 and folate deficiencies. Hcy is implicated in both inflammatory processes and bone remodeling [39,52]. Some in vitro studies demonstrated that Hcy directly promotes osteoclast differentiation and survival, inhibits osteoblast activity [53,54] and influences bone matrix by interfering with collagen cross-links [55]. Previous studies also suggest that Hcy may exert detrimental effects on bone through a decrease of blood flow in the bone tissue and an increase of metalloproteinases, which degrade extracellular bone matrix [39,56]. An increased Hcy serum level might therefore represent an independent risk factor for osteoporosis. Moreover, Hcy also enhances inflammatory processes, which in turn induce a skeletal demineralization due to the activation of bone resorption, as well as an inhibition of new bone formation, thus affecting bone strength [42]. Vitamin B12 and folate are involved in both Hcy metabolism and bone turnover, acting on bone via direct and indirect effects [45]. In fact, in addition to the regulation of Hcy metabolism, these nutrients also modulate bone cell function; in particular, they stimulate osteoblasts whereas their deficiency induces osteoclast activity [22].

Our results show an overall inverse correlation between Hcy levels and T-score values in the total sample of postmenopausal women, but this significant inverse correlation is lost in the group of 34 (13%) women with fractures. This could depend on the small sample size of fractured patients. Interestingly, fractured patients with normal BMD all showed elevated serum Hcy levels, suggesting that hyperhomocysteinemia could induce bone fragility and increased risk of fractures also through mechanisms impacting on the composition of bone matrix proteins and not only on the BMD [51]. This hypothesis, according to other reports supporting direct effects of Hcy on collagen post-translational modifications that are independent of bone mineral characteristics [57,58], could partly explain our observations of lack of direct correlation between Hcy and BMD in fractured patients. There are a lot of contributing factors determining bone quality, such as inflammation, vitamin deficiencies, lifestyle, genetic and environmental factors, which differently act in each patient, interacting variously with each other.

This study has some limitations. One of these is the relatively small size of the sample studied, which increases the uncertainty of some results. For example, the low statistical significance of the difference between serum BAP levels in women with decreased BMD and those in women with normal BMD, the absence of statistical significant difference in BAP levels between women with normal and high Hcy, as well as the lack of a highly significant negative correlation between BMD and homocysteine levels in fractured patients could depend on the low sample size and the wide range of variability of the values. Another limitation is the lack of diet analysis in studied women. Since the primary goal of the study was to verify the association between hyperhomocysteinemia, inflammation and decrease in BMD, rather than the search for the causes of increased homocysteine in the blood, dietary habits of the women studied were not in depth investigated. From a nutritional point of view, only serum levels of vitamin D, vitamin B12 and folate have been investigated, which, together with the MTHFR C677T mutation, may influence serum homocysteine levels, as well as BMD. Further studies with larger samples are needed to evaluate the influence of lifestyle habits and in particular diet on homocysteine and T-score levels of postmenopausal women. The high socio-sanitary burden of osteoporosis and the need for cost-effective prevention programs could justify planning future researches on a larger scale.

## 5. Conclusions

In summary, our study suggests a significant association between Hcy and postmenopausal osteoporosis. Notwithstanding the many efforts to effectively diagnose and prevent osteoporosis occurrence [2,59], the results are not fully satisfactory. An imbalance in Hcy metabolism may contribute to a derangement of bone remodeling, favoring the onset of osteoporosis. The regulation of Hcy overproduction and the modulation of the inflammatory substrate that characterizes menopause may represent additional therapeutic approaches for osteoporosis prevention and control.

## Figures and Tables

**Table 1:** Vitamin D, folate and percentage of MTHFRC6775 in women with hyperhomocysteinemia and in women with normal homocysteine level.

Laboratory findings	Women with Serum HcyConcentration > 12 μmol/L (n. 147)	Women with Serum HcyConcentration < 12 μmol/L (n. 105)	
	Means ± Standard Deviations		*p* values
**ESR**	23.54 mm/h ± 13.77	16.65 mm/h ± 9.78	*p* < 0.001
**CRP**	7.46 mg/L ± 7.44	3.09 mg/L ± 2.98	*p* < 0.001
**OC**	20.80ng/mL ± 8.01	25.13 ng/mL ± 7.54	*p* < 0.001
**CTX**	451.64 pg/mL ± 237.05	317.77 pg/mL ± 190.67	*p* < 0.001
**BAP**	11.20 µg/L ± 4.74	11.78 µg/L ± 5.56	ns
**VIT B 12**	376.16 pg/mL ± 147.20	455.79 pg/mL ± 167.50	*p* < 0.001
**FOLATE**	5.69 ng/mL ± 3.13	9.16 ng/mL ± 4.34	*p* < 0.001
**VIT D**	22.03 ng/mL ± 9.56	27.84 ng/mL ± 10.95	*p* < 0.001
**MTHFR C677T**	57.14%	33%	*p* < 0.001
